# Surgical treatment for pancreatic cystic lesions—implications from the multi-center and prospective German StuDoQ|Pancreas registry

**DOI:** 10.1007/s00423-022-02740-0

**Published:** 2023-01-14

**Authors:** Jonas Henn, Patricia K. Wyzlic, Irene Esposito, Alexander Semaan, Vittorio Branchi, Carsten Klinger, Heinz J. Buhr, Ulrich F. Wellner, Tobias Keck, Philipp Lingohr, Tim R. Glowka, Steffen Manekeller, Jörg C. Kalff, Hanno Matthaei, the StuDoQ|Pancreas Study Group

**Affiliations:** 1 Department of General, Visceral, Thoracic and Vascular Surgery, University Hospital Bonn, Bonn, Germany; 2 Institute of Pathology, Heinrich-Heine University, Düsseldorf, Germany; 3 German Society of General and Visceral Surgery (DGAV), Berlin, Germany; 4 Department of Surgery, UKSH Campus Lübeck, Lübeck, Germany

**Keywords:** Pancreas, Surgical Oncology, Pancreatic Cysts, National Registry

## Abstract

**Purpose:**

The detection of pancreatic cystic lesions (PCL) causes uncertainty for physicians and patients, and international guidelines are based on low evidence. The extent and perioperative risk of resections of PCL in Germany needs comparison with these guidelines to highlight controversies and derive recommendations.

**Methods:**

Clinical data of 1137 patients who underwent surgery for PCL between 2014 and 2019 were retrieved from the German StuDoQ|Pancreas registry. Relevant features for preoperative evaluation and predictive factors for adverse outcomes were statistically identified.

**Results:**

Patients with intraductal papillary mucinous neoplasms (IPMN) represented the largest PCL subgroup (*N* = 689; 60.6%) while other entities (mucinous cystic neoplasms (MCN), serous cystic neoplasms (SCN), neuroendocrine tumors, pseudocysts) were less frequently resected. Symptoms of pancreatitis were associated with IPMN (*OR*, 1.8; *P* = 0.012) and pseudocysts (*OR*, 4.78; *P* < 0.001), but likewise lowered the likelihood of MCN (*OR*, 0.49; *P* = 0.046) and SCN (*OR*, 0.15, *P* = 0.002). A total of 639 (57.2%) patients received endoscopic ultrasound before resection, as recommended by guidelines. Malignancy was histologically confirmed in 137 patients (12.0%), while jaundice (*OR*, 5.1; *P* < 0.001) and weight loss (*OR*, 2.0; *P* = 0.002) were independent predictors. Most resections were performed by open surgery (*N* = 847, 74.5%), while distal lesions were in majority treated using minimally invasive approaches (*P* < 0.001). Severe morbidity was 28.4% (*N* = 323) and 30d mortality was 2.6% (*N* = 29). Increased age (*P* = 0.004), higher BMI (*P* = 0.002), liver cirrhosis (*P* < 0.001), and esophageal varices (*P* = 0.002) were independent risk factors for 30d mortality.

**Conclusion:**

With respect to unclear findings frequently present in PCL, diagnostic means recommended in guidelines should always be considered in the preoperative phase. The therapy of PCL should be decided upon in the light of patient-specific factors, and the surgical strategy needs to be adapted accordingly.

**Supplementary Information:**

The online version contains supplementary material available at 10.1007/s00423-022-02740-0.

## Introduction

High-resolution cross-sectional imaging has become increasingly important in modern medicine and radiologic studies for various reasons often include the pancreas due to its anatomic location. Thus, the detection of pancreatic cystic lesions (PCL) has increased dramatically over the recent decades culminating in a prevalence of approximately 15% in the normal population using magnetic resonance imaging (MRI) [1]. Since PCL comprise a plethora of histologic lesions with partially severe clinical significance, a further diagnostic assessment is demanded [2]. Interdisciplinary teams must essentially distinguish non-neoplastic lesions (e.g., pseudocysts) from neoplastic PCL, which harbor the potential for malignant transformation [3]. Specifically, intraductal papillary mucinous neoplasms (IPMN) and mucinous cystic neoplasms (MCN) share a significant malignant potential and represent the most frequent neoplastic cysts [3–5]. Serous cystic neoplasms (SCN) are less common and virtually always benign [6]. Furthermore, cystic variants of neuroendocrine tumors (cNET) are rarely detected in the pancreas and tend to be clinically less aggressive compared to their solid counterparts [7].

Unfortunately, even the latest cross-sectional imaging techniques cannot replace the gold standard of a thorough histopathologic analysis in finding the correct diagnosis in a PCL. Additionally, these radiologic modalities fail to confidently predict the malignant progression of PCL reflected by positive prediction rates ranging from 71–80% for computed tomography (CT) and 55–76% for MRI scans [8]. Endoscopic ultrasound (EUS) may provide additional diagnostic information and the opportunity of fine needle aspiration (FNA) for harvesting tissue specimens from the cystic wall or cyst fluid aspirates for emerging molecular tests [9]. Especially, the assessment of specific genetic alterations has shown promising implications for a differentiation among entities, and their further investigation will likely improve patient care [10–12]. Besides those recent advances, available treatment algorithms are hardly backed by larger prospective observational studies. Instead, current guidelines are mainly based on expert consensus thus lacking statistical evidence [8, 13]. However, a recently conducted survey revealed marked differences in PCL management even among international experts further stressing the need for standardization [14]. Although the outcome after pancreatic resection has improved over the last decades, morbidity and mortality rates remain on the highest levels within abdominal surgery [15]. Therefore, in addition to the diagnostic assessment, the surgical strategy plays a central role. National registries such as the Study-, Documentation, and Quality- Center (StuDoQ) of the German Association for General- and Visceral- Surgery (DGAV) are promising tools in generating evidence-based strategies on a multicenter effort [16]. Thus, this study aims to describe the status of surgical therapy of PCL in Germany against the background of international guidelines: which operations are performed? What risks does this pose to patients? What recommendations for action can be derived from this?

## Materials and methods

### Patient cohort

Data was retrieved from the multicenter StuDoQ|Pancreas registry by the German Society for General and Visceral Surgery (DGAV). This prospectively maintained database was established in 2013 recording detailed information from approximately 10–20% of all patients who undergo pancreatic resection in Germany [17]. Patients with histopathologically confirmed PCL between 2014 and 2019 were enrolled comprising the most diagnosed cystic entities, namely IPMN, MCN, SCN, cNET, and pseudocysts. Patients signed informed consent for anonymized participation in the registry, and the Ethics Committee, University of Bonn, Germany, approved the present study (#498/20).

### Baseline characteristics

The StuDoQ|Pancreas registry records a broad spectrum of pre-, intra-, and postoperative data while the query was adapted to the present research project. Basic demographic and clinical information included age, gender, body mass index (BMI), American Society of Anesthesiologists Score (ASA), and smoking status [18]. Medical history comprised information on intake of immunosuppressive drugs or corticosteroids, symptoms at diagnosis (i.e., abdominal pain, nausea, emesis, jaundice, sepsis, and weight loss), alcohol abuse, liver cirrhosis, stented common bile duct, esophageal varices, ascites, symptoms of acute (AP) and chronic pancreatitis (CP), diabetes mellitus (DM), and insulin dependency. Furthermore, the utilization of diagnostic modalities such as abdominal sonography, CT, MRI, EUS, and/or endoscopic retrograde cholangiopancreatography (ERCP) was recorded, including information on complications associated with interventional imaging modalities. Intraoperative information comprised type of access (open vs. minimally invasive surgery (MIS)), need for conversion to open surgery, type of resection, and extent of lymph node dissection (LND) according to the German S3-Guidelines [19]. MIS procedures included laparoscopic (assisted) and robotic (assisted) procedures, while no data was available on how many robotic procedures were performed. PCL were histologically categorized according to international consensus recommendations while associated malignant tumors were classified respecting the 8th Edition of the UICC TNM Classification of Malignant Tumors [20, 21]. PCL were defined as “malignant” whenever an associated invasive carcinoma was present, or a high-grade (G2-3) cNET was histologically diagnosed. The Clavien-Dindo classification was used to categorize postoperative complications, while a score > 2 was defined as “severe morbidity” [22]. The following pancreatectomy-specific complications were analyzed separately: pancreatic fistula (PF), surgical site infection (SSI), delayed gastric emptying (DGE), post pancreatectomy hemorrhage (PPH), and need for antidiabetic treatment [23–25]. Overall hospitalization time, days treated at intensive care unit (ICU), and 30d mortality were also reported.

### Statistical analysis

Descriptive and inferential statistics were applied in data analyses using R (R Foundation for Statistical Computing, Vienna, Austria) and RStudio version 1.3.1093 (RStudio, Inc., Boston, USA). To avoid multiple testing errors, we conducted an explorative analysis. Herein, new hypotheses are generated, and positive results need to be confirmed in further, more targeted studies. All reported *P*-values were two-sided, and results with *P* < 0.05 were included into further evaluation. Intergroup differences were calculated using Fisher’s exact test for categorical variables and Student’s *t*-test for continuous variables (applying the central limit theorem). For risk factor analysis, parameters were plotted versus the outcome in a multivariate linear or binary logistic regression model. Whenever applicable, results were presented with an odds ratio (*OR*), the 95% confidence interval (*CI95*), and regarding *P*-value.

## Results

### Clinical presentation and preoperative assessment

A total of 1190 patients with PCL were operated on between 2014 and 2019 at German centers involved in the prospective StuDoQ|Pancreas registry. Two patients were excluded due to insufficient datasets, and 51 patients were not integrated because they underwent non-resection procedures (e.g., drainage operation). Clinical characteristics of the remaining 1137 patients are summarized in Table 1. Sufficient information on preoperative imaging was available in 1104 patients (97.1%). Modalities applied in those included abdominal sonography (*N* = 609/1104, 55.2%), CT (*N* = 788/1104, 71.4%), MRI (*N* = 631/1104, 57.2%), and EUS (*N* = 639/1104, 57.9%). While 251 of 1104 patients (22.7%) received CT + EUS, 159 (14.4%) received MRI + EUS, and 196 (17.8%) had CT + MRI + EUS. In 153 patients (13.9%), an ERCP was conducted preoperatively, mainly for cholestasis and stent placement. A small subset experienced post-ERCP complications (*N* = 10/153, 6.5%) including acute pancreatitis (*N* = 8/153, 5.2%) and bowel perforation (*N* = 2/158, 1.3%).

**Table 1:** Baseline characteristics of included patients, divided by histological confirmation of malignancy within the resected PCL

	All		Benign		Malignant		*P*
All (*n*, %)	1137	100	1000	88	137	12	-
Sex (*n*, %)							
Female (*n*, %)	663	58	591	59	72	53	0.166
Male (*n*, %)	474	42	409	41	65	47	0.166
Age (median *y*, IQR)	68	58–75	68	58–75	71	61–76	0.006
BMI (median kg/m^2^, IQR)	25	23–28	25	23–28	24	22–27	0.034
ASA							
I (*n*, %)	77	7	70	7	7	5	0.474
II (*n*, %)	590	52	531	53	59	43	0.029
III (*n*, %)	462	41	391	39	71	52	0.005
IV (*n*, %)	8	1	8	1	0	0	0.606
Smoker (*n*, %)	153	13	135	14	18	13	1
Immunosuppression (*n*, %)	10	1	10	1	0	0	0.619
Corticosteroids (*n*, %)	20	2	18	2	2	2	1
Any symptoms (*n*, %)	507	45	435	44	72	53	0.054
Abdominal pain (*n*, %)	378	33	337	34	41	30	0.439
Nausea (*n*, %)	136	12	118	12	18	13	0.673
Emesis (*n*, %)	38	3	33	3	5	4	0.800
Jaundice (*n*, %)	35	3	21	2	14	10	0.018
Sepsis (*n*, %)	6	1	5	1	1	1	0.538
Weight loss (*n*, %)	133	12	101	10	32	23	0.030
Liver specific (*n*, %)	130	11	110	11	20	15	0.251
Alcohol (*n*, %)	45	4	41	4	4	3	0.644
Cirrhosis (*n*, %)	22	2	20	2	2	1	1
Esophageal varices (*n*, %)	6	1	6	1	0	0	1
Ascites (*n*, %)	12	1	10	1	2	1	0.647
Stented CBD (*n*, %)	65	6	49	5	16	12	0.005
Pancreas specific (*n*, %)	424	37	366	37	58	42	0.221
Symptoms of AP (*n*, %)	119	10	105	11	14	10	1
Symptoms of CP (*n*, %)	130	11	117	12	13	9	0.566
DM (*n*, %)	264	23	218	22	46	34	0.003
IDDM (*n*, %)	117	10	97	10	20	15	0.097
Type Of resection							
PD (*n*, %)	476	42	417	42	59	43	0.782
DP (*n*, %)	476	42	435	44	41	30	0.003
TP (*n*, %)	115	10	83	8	32	23	< 0.001
Atypical (*n*, %)	70	6	65	7	5	4	0.255
Entity							
IPMN (*n*, %)	689	61	599	60	90	66	0.226
MCN (*n*, %)	172	15	140	14	32	23	0.007
SCN (*n*, %)	161	14	159	16	2	1	< 0.001
cNET (*n*, %)	33	3	20	2	13	9	0.044
Pseudocyst (*n*, %)	82	7	82	8	0	0	0.028

*BMI* body mass index, *ASA* American Society of Anesthesiologists Score, *CBD* common bile duct, *AP* acute pancreatitis, *CP* chronic pancreatitis, *DM* diabetes mellitus, *CT* computed tomography, *MRI* magnetic resonance imaging, *EUS* endoscopic ultrasound, *ERCP* endoscopic retrograde cholangiopancreatography, *PD* pancreatoduodenectomy, *DP* distal pancreatectomy, *TP* total pancreatectomy, *IPMN* intraductal papillary mucinous neoplasm, *MCN* mucinous cystic neoplasm, *SCN* serous cystic neoplasm, *cNET* cystic neuroendocrine tumor, *ICU* intensive care unit

### Surgical approach

For further analysis, resections were divided into pancreatoduodenectomy (PD), distal pancreatectomy (DP), and total pancreatectomy (TP). Any resection of parenchyma not following this classification was marked as “atypical.” While PD (*N* = 476, 41.9%) and DP (*N* = 476, 41.9%) were predominant and equally distributed, TP (*N* = 115, 10.1%) and atypical resections (*N* = 70, 6.2%) were performed less commonly. In comparison to all other resected PCL, IPMN were more often treated with PD (*P* < 0.001) and TP (*P* < 0.001), while DP was less commonly performed (*P* < 0.001). TP was unusual in MCN and SCN (*P* = 0.009 and *P* < 0.001), which were more often resected with DP (*P* < 0.001 and *P* < 0.031). Most resections were performed by conventional “open” surgery (*N* = 847, 74.5%), while a MIS (including robotic surgery) approach was attempted in 290 patients (25.5%). Of those, every fifth patient required conversion to open surgery (*N* = 58, 20.0%). Compared to all resections, PD and TP were predominantly conducted through open surgery (*P* < 0.001 and *P* < 0.001), and DP was notably more often performed using MIS (*P* < 0.001). Most patients received standard loco-regional LND (*N* = 738, 64.9%) while a small subset underwent extended LND (*N* = 52, 4.6%). Standard and extensive LND were less commonly carried out in patients approached with MIS techniques (*P* = 0.001 and *P* = 0.002). Furthermore, PD and TP were usually combined with standard LND (*P* < 0.001 and *P* = 0.001), while DP and atypical resections led to less LND overall (*P* < 0.001 and *P* < 0.001). Standard LND was more often performed for IPMN (*P* < 0.001) and less frequently when final histology revealed MCN and pseudocysts (*P* = 0.030 and *P* < 0.001).

### PCL entities

IPMN was the most frequently resected PCL (*N* = 689, 60.6%), followed by MCN (*N* = 172, 15.1%), SCN (*N* = 161, 14.2%), pseudocysts (*N* = 82, 7.2%), and cNET (*N* = 33, 2.9%). Table 2 presents statistically significant factors of a specific cystic entity in comparison with the remaining PCL entities as well as parameters that indicate malignancy. While older patients were particularly more often diagnosed with IPMN (*OR*, 1.06; *CI95*, 1.05–1.07; *P* < 0.001), younger age was a predictive factor for MCN (*OR*, 0.96; *CI95*, 0.94–0.97; *P* < 0.001), cNET (*OR*, 0.96; *CI95*, 0.93–0.98; *P* = 0.001) and pseudocysts (*OR*, 0.96; *CI95*, 0.94–0.98; *P* < 0.001). Furthermore, the male sex was a prognostic factor for IPMN (*OR*, 1.5; *CI95*, 1.14–1.97; *P* = 0.005), whereas the female sex was strongly associated with MCN (*OR*, 0.60; *CI95*, 0.40–0.89; *P* = 0.012) and SCN (*OR*, 0.63; *CI95*, 0.43–0.92; *P* = 0.018). Finally, symptoms of pancreatitis showed predictive potential: on the one hand, AP was oftentimes present in patients with IPMN (*OR*, 1.84; *CI95*, 1.15–2.98; *P* = 0.012) and CP in those with pseudocysts (*OR*, 4.78; *CI95*, 2.65–8.55; *P* < 0.001). On the other hand, patients with MCN lacked symptoms of CP (*OR*, 0.49; *CI95*, 0.23–0.95; *P* = 0.046), and patients harboring SCN were less often afflicted by symptoms of both AP and CP (*OR*, 0.15; *CI95*, 0.04–0.42; *P* = 0.002 and *OR*, 0.26; *CI95*, 0.09–0.61; *P* = 0.006). Alcohol abuse elevated the risk for pseudocysts (*OR*, 4.15; *CI95*, 1.75–9.37; *P* < 0.001). Pathology confirmed 137 (12.0%) malignant PCL. While SCN were less likely to be malignant (1.2%, *P* < 0.001), MCN showed an increased rate of malignancy, when compared to other PCL (18.6%, *P* = 0.007). Jaundice (*OR* = 4.03, *CI95* 1.59–10.10, *P* = 0.003) and weight loss (*OR*, 2.16; *CI95*, 1.12–4.14; *P* = 0.020) were individual risk factors for the presence of a malignant PCL.

**Table 2:** Results of multivariate analysis for possible distinguishing factors between PCL entities

	Probability	*OR* (*CI95*)	*P*
Malignancy			
Jaundice	+	4.03 (1.59–10.10)	0.003
Weight loss	+	2.16 (1.12–4.14)	0.020
IPMN			
Older Age	+	1.06 (1.05–1.07)	< 0.001
Male Sex	+	1.49 (1.14–1.97)	0.004
Nausea	−	0.50 (0.30–0.81)	0.005
Stented CBD	+	2.05 (1.05–4.17)	0.040
Symptoms of AP	+	1.84 (1.15–2.98)	0.012
MCN			
Older Age	−	0.96 (0.94–0.97)	< 0.001
Male Sex	−	0.60 (0.40–0.89)	0.012
Symptoms of CP	−	0.49 (0.23–0.95)	0.046
SCN			
Male Sex	−	0.63 (0.43–0.92)	0.018
Smoking	−	0.42 (0.20–0.79)	0.012
Symptoms of AP	−	0.15 (0.04–0.42)	0.002
Symptoms of CP	−	0.26 (0.09–0.61)	0.006
cNET			
Older Age	−	0.96 (0.93–0.98)	< 0.001
Pseudocyst			
Older Age	−	0.96 (0.94–0.98)	< 0.001
Smoking	+	2.46 (1.34–4.41)	0.003
Alcohol	+	4.15 (1.75–9.37)	< 0.001
Symptoms of CP	+	4.78 (2.65–8.55)	< 0.001

*OR* Odds Ratio, *CI95* 95% confidence interval, *PCL* pancreatic cystic lesion, *PDAC* pancreatic ductal adenocarcinoma, *CBD* common bile duct, *AP* acute pancreatitis, *CP* chronic pancreatitis, *IPMN* intraductal papillary mucinous neoplasm, *MCN* mucinous cystic neoplasm, *SCN* serous cystic neoplasm, *cNET* cystic neuroendocrine tumor

### Postoperative outcome

The postoperative outcome, divided by symptomatic and incidental PCL, is summarized in Table 3. Patients were hospitalized for a median of 15 days (IQR 11–23) and needed intensive care unit (ICU) treatment for a median of 2 days (IQR 1–4). Overall morbidity was 59.5% (*N* = 676, Clavien-Dindo > 0). While just over half of those (*N* = 353/676, 52.2%) needed only minor treatment (i.e., medication, nutrition, minor wound treatment), the remaining (*N* = 323/676, 47.8%) suffered from severe complications, including life-threatening and lethal conditions. In detail, one-fifth of patients suffered from grade B/C PF (*N* = 213, 18.7%), while every sixth patient (*N* = 180/1137, 15.8%) showed postoperative DGE. About a tenth of all patients suffered from PPH (*N* = 102, 9.0%), and the overall incidence for SSI was low (*N* = 72, 6.3%). Three-quarters (*N* = 873, 76.8%) of patients showed no signs of DM preoperatively, of which only 12.8% (*N* = 112/873) needed insulin treatment postoperatively. Nearly every fourth patient with preoperatively known IDDM did not require any further insulin treatment postoperatively (*N* = 27/117, 23.1%). Independent risk factors for morbidity and complications are shown in Table 4. The overall 30d mortality was 2.6% (*N* = 29), whereas older age, higher BMI, smoking, cirrhosis, varices, and symptoms of CP independently increased the risk for a lethal course (for details see Table 4).

**Table 3:** Postoperative Outcome, divided by symptomatic and incidental PCL

	All			Symptomatic			Incidental				*P*
All (*n*, %)	1 137	100		507	45		630		55		-
Severe morbidity (CD > 2) (*n*, %)	323	28		138	43		185		57		0.428
PF (grade B/C) (*n*, %)	213	19		84	39		129		61		0.108
DGE (*n*, %)	180	16		79	44		101		56		0.870
PPH (*n*, %)	102	9		44	43		58		57		0.835
Antidiabetic treatment* (*n*, %)	129	11		55	43		74		57		0.707
SSI (*n*, %)	72	6		35	49		37		51		0.541
Stay median *d*, IQR	15	11	23	15	11	22		15	11	23	0.201
ICU median *d*, IQR	2	1	4	2	1	4		2	1	4	0.783
30d mortality (*n*, %)	29	3		14	48		15		52		0.709

*CD* Clavien-Dindo, *PF* pancreatic fistula, *DGE* delayed gastric emptying, *PPH* post pancreatectomy hemorrhage, *SSI* surgical site infection, *ICU* intensive care unit

^*^For patients without preoperative known DM

**Table 4:** Independent predictive factors on morbidity, complications, and 30d mortality, as identified by multivariate analysis. *OR* is shown for categorical variables and estimate is shown for continuous variables, respectively

	Probability	*OR*/Estimate (*CI95*)	*P*
Severe morbidity (CD < 2)			
Age	+	0.934 (0.076–1.814)	0.035
ASA 4	+	2.523 (0.661–5.506)	0.024
Corticosteroids	+	1.456 (0.515–2.448)	0.003
PD	+	0.879 (0.253–1.565)	0.008
PF			
BMI	+	2.173 (0.741–3.605)	0.003
Corticosteroids	+	1.322 (0.298–2.349)	0.010
DGE			
MIS	−	− 0.626 (− 1.283 to 0.030)	0.049
Conversion	+	1.414 (0.549–2.277)	0.001
PD	+	1.566 (0.614–2.798)	0.004
TP	+	1.681 (0.634–2.970)	0.004
PPH			
Age	+	1.581 (0.132–3.113)	0.037
Varices	+	2.633 (0.484–4.637)	0.009
Diabetes	−	− 0.860 (− 1.718 to 0.133)	0.031
Antidiabetic treatment*			
Age	+	3.049 (1.473–4.722)	< 0.001
BMI	+	3.585 (1.409–5.789)	0.001
Smoker	+	0.848 (0.214–1.455)	0.007
TP	+	5.277 (3.805–7.264)	< 0.001
SSI			
BMI	+	2.475 (0.266–4.608)	0.025
Nausea	+	0.799 (0.043–1.501)	0.031
Stay			
Age	+	6.583 (1.128–12.038)	0.018
ASA 4	+	14.299 (2.835–25.763)	0.015
BMI	+	10.857 (2.364–19.351)	0.012
Corticosteroids	+	7.716 (0.684–14.749)	0.032
Diabetes	−	− 2.807 (− 5.612 to 0.002)	0.050
MIS	−	− 3.138 (− 5.651 to 0.625)	0.014
PD	+	4.088 (0.065–8.110)	0.046
TP	+	6.863 (2.069–11.658)	0.005
ICU			
Age	+	3.623 (1.009–6.237)	0.007
ASA 4	+	16.170 (10.677–21.663)	< 0.001
BMI	+	8.940 (4.870–13.010)	< 0.001
Cirrhosis	+	4.329 (1.080–7.578)	0.009
Conversion	+	2.253 (0.105–4.401)	0.040
PD	+	2.199 (0.271–4.126)	0.025
TP	+	3.374 (1.076–5.671)	0.004
30d mortality			
Age	+	6.443 (2.778–10.655)	0.001
BMI	+	8.361 (4.428–12.534)	< 0.001
Smoker	+	1.212 (0.019–2.315)	0.036
Cirrhosis	+	3.351 (1.702–5.004)	< 0.001
Varices	+	3.938 (1.305–6.595)	0.002
CP	+	1.285 (− 0.079 to 2.511)	0.047

*OR* odds ratio, *CI95* 95% confidence interval, *CD* Clavien-Dindo, *ASA* American Society of Anesthesiologists Score, *PD* pancreatoduodenectomy, *PF* pancreatic fistula, *BMI* body mass index, *DGE* delayed gastric emptying, *MIS* minimally invasive surgery, *TP* total pancreatectomy, *PPH* post pancreatectomy hemorrhage, *SSI* surgical site infection, *CBD* common bile duct, *ICU* intensive care unit, *CP* chronic pancreatitis

^*^For patients without preoperative known DM

## Discussion

The detection of PCL upon cross-sectional imaging is rising and leaves clinicians with a diagnostic dilemma. Careful considerations must be undertaken to balance potentially harmful surgical overtreatment of non-malignant PCL against the threat of malignant progression during a watchful waiting strategy. The lack of valid treatment algorithms for evidence-based decision-making in PCL as well as conflicting opinions—even among experts—continues to create uncertainty in both patients and physicians. Thus, adequately powered multi-center studies are urgently needed to improve patient care in this common clinical conundrum [2, 14]. Herein, we present experience from (one of) the largest recent national multi-center cohorts of patients with resected PCL listed in the German StuDoQ|Pancreas registry. Our intention was to provide additional evidence for prevailing questions in the surgical treatment of PCL: How can we determine the indication for resection? Are the respective patients clinically suitable for pancreatic surgery? What is the appropriate surgical approach?

The fact that our study revealed an overall low rate of PCL-associated malignancy and the information that merely half of patients suffered from preoperative symptoms clearly raises the question of what reasons led to surgery in the remaining individuals. Despite a comprehensive dataset available for every patient in the registry, the indication for surgery is, unfortunately, not yet an item of the digital documentation form. It can only be assumed that reasons for surgery (other than malignancy and symptoms) included endoscopically proven high-grade dysplasia in mucinous cysts (IPMN and MCN), increase in cyst size, or the presence of significant mural nodules within preoperative imaging. For the prediction of a cystic entity and to gather information of a possible malignant potential, a combination of various diagnostic categories is generally recommended [26]. Demographic and clinical data may provide preliminary suspicion of a specific PCL subtype. Accordingly, we identified age and sex as helpful features in this respect: male patients are more likely to suffer from IPMN than from SCN or MCN, while harboring an MCN was, as expected, characteristic for women [3]. Whereas history of pancreatitis was confirmed as a well-known risk factor for the presence of pseudocysts, symptoms of pancreatitis were also correlated with IPMN in our cohort [27]. In contrast, the presence of those symptoms lowered the probability of harboring a MCN or SCN. Preoperative jaundice and weight loss have been previously associated with IPMN with an invasive component [28]. Interestingly, this coherence applied to any “malignant” PCL, independent of histology in our series. Although smoking was no independent predictor for malignancy in our cohort, it remains a relevant clinical factor for its likely role in the accelerated malignant progression of IPMN [29]. Aside from demographic and clinical parameters, radiological features serve as the second important source of indicators for PCL entity. In fact, the parameters of PCL localization, size, configuration, and presence of lymphadenopathy are particularly helpful in the preoperative assessment of suspicious cysts [28]. Unfortunately, such information is yet missing in the registry due to the primarily surgical focus of the Studoq registry. Although, recent PCL guidelines recommend EUS to detect features of malignancy such as suspicious lymph nodes or cyst wall thickening, around 40% of operated patients did not receive EUS [8, 13]. Besides the opportunity for closeup visual assessment, EUS provides the opportunity to safely obtain cyst fluid or to harvest a tissue specimen via FNA for further analysis [9]. In detail, cytology, protein markers, and genetic alterations were successfully used to improve the detection of high-grade dysplasia [10, 30, 31]. The integration of relevant factors from clinical, radiological, and molecular data seems crucial for the future preoperative PCL workup while experts agree that meticulously planned multi-center studies are inevitable to create the urgently needed therapeutic evidence in PCL [14, 32]. Besides their superiority in helping to refine surgical quality, nationwide registries can also provide the framework for the implementation of state-of-the-art diagnostic standards. Consequently, datasets will continue to grow (aka *big data*), and therefore, the combination with modern data technologies may provide unprecedented opportunities for evidence-based medicine in PCL [33]. First exciting approaches to prevent unnecessary PCL resections with the help of machine learning have only recently been published, and we are convinced this promising field justifies further scientific dedication [34]. Given the sometimes-unclear indication, we recommend an increased use of helpful diagnostic means as recommended in current guidelines [8, 13].

As soon as diagnostic studies indicate the oncological benefit of resecting a PCL, physicians must carefully assess a patients’ clinical condition to decide if he or she is suitable for pancreatic surgery. In line with recently published data, our PCL cohort comprised mostly female patients that were middle-aged and slightly overweight [35]. Not surprisingly, a large fraction of patients had preexisting comorbidities adding to the risk for adverse perioperative outcomes. Not surprisingly, our analysis identified older age as an independent risk factor for severe morbidity, more pancreatectomy-related complications, longer stays, longer ICU treatment, and mortality. The median length of hospital stay in our cohort is in line with that one reported in other pancreatectomy studies from Germany. The marked differences in hospital stay among different countries are known and described elsewhere [16]. Compared to other recent studies, the overall 30d mortality was similar in our PCL cohort whereas higher age, impaired liver function, and BMI were both confirmed as independent risk factors [36]. For preoperative factors, a recent monocentric work did not find a difference in postoperative outcome between obese and non-obese patients. In contrast, a higher BMI in our cohort appears to significantly increase the risk for PF, need for antidiabetic treatment, SSI, longer stay, longer ICU treatment, and mortality. While the controversial results are certainly partly due to different methodologies, the role of obesity as a risk factor for adverse outcomes has not yet been conclusively established in PCL [37]. Corticosteroid use is known to increase the incidence of adverse events such as SSI and mortality, and this effect was clearly confirmed for our PCL cohort [38]. Liver cirrhosis is related to portal hypertension as well as coagulopathies, and affected patients showed an increased risk for adverse events and may therefore provoke more ICU-bound complication management as well as mortality. Warnick et al. came to a similar conclusion in a case–control study and recommended that only patients with CHILD A cirrhosis should undergo pancreatic resection [39]. Most patients operated on did not need antidiabetic treatment in the postoperative course while elderly and obese patients were overrepresented in the group of individuals who required medication to normalize their serum glucose levels. Interestingly, a relevant subset of patients with preoperatively documented IDDM needed no antidiabetic treatment in the postoperative course. Kang et al. made similar observations and proposed changes in insulin secretion and reconstruction-induced anatomical changes as the most probable associated cause. The authors even suggested that type of reconstruction may impact the DM-related outcome, why future studies need to further explore this therapeutic option [40]. Overall, if patients present with older age, obesity, and/or impaired liver function, special caution is advised before recommending a resection, and possible prehabilitation actions should be evaluated [41].

Interestingly, international guidelines provide no specific technical guidance for PCL (aside from drainage operations in pseudocysts). Usually, oncological pancreatectomy with standard lymphadenectomy is recommended [8, 13]. As observed in our cohort, a vast majority of PCL resections were performed in a conventional manner or needed conversion to open surgery, when started laparoscopically, while the individual underlying causes are not documented. The use of MIS reduced the risk for DGE and led to shorter hospital stays in our cohort. Likewise, the need for conversion increased the risk for DGE and longer ICU treatment. In general, possible benefits associated with MIS in pancreatectomy are well established [42], and Klompmaker et al. in their recent multi-center study could prove that MIS pancreatic resection is comparable to open pancreatectomy in terms of morbidity and mortality, while a robotic-assisted approach could reduce conversion rates substantially [43]. Participating surgeons mainly chose oncological resections (e.g., DP, PD, TP) for treating PCL. Recently, parenchyma-sparing procedures (i.e., atypical resections) were evaluated for resecting IPMN aiming at the possible improvement of long-term outcome. Although a higher morbidity has been linked to less-radical operations in PCL, atypical resections did neither cause additional morbidity nor an increase in mortality in our cohort [44, 45]. Given the overall low rate of malignant PCL, treating physicians should consider the use of parenchyma-sparing resections and, if appropriate, discuss them openly with their patients. Intraoperatively, LND is a key task in oncological surgery to remove the entire tumor burden and to allow for precise tumor assessment to settle for appropriate oncological management [42]. For pancreatic cancer, standard LND represents a balance between higher yield for an improved outcome and the risk of increased morbidity caused by excessive LND [46]. The current European consensus recommends standard LND for all IPMN and MCN resections, while other entities are not addressed, and a respective statement is entirely missing in the ACG guidelines [8, 13]. Uncertainties in preoperative estimation of PCL histology and the presence of malignancy make an application of LND guidelines challenging, why intraoperative decision-making is of crucial importance. Herein, frozen section analysis is helpful when proving malignancy, while negative results (i.e., frozen analysis shows no cancer or unclear histology) harbor a noteworthy risk for missing entities actually demanding LND [47]. Surgeons in the present study usually performed standard LND when resecting a PCL, and LND had no independent effect on postoperative outcomes. However, for unrecorded causes, we observed a substantially lower rate of standard LND in MIS procedures. This might be caused by the technical challenge to conduct a neat LND in a minimally invasive manner. Once the surgical team decides to resect a PCL, there appears to be little reason not to perform standard LND, and it should therefore be performed appropriately. Ultimately, robotic techniques might be able to guarantee better tumor assessment as well as performing a sufficient LND through optimized visualization and enhanced dexterity [48]. Surgical teams should discuss the possibility of an MIS approach with PCL patients because of potential benefits. Herein, careful consideration must be given to patient-specific factors, and future studies will show whether robotic resection is the most appropriate technique.

Despite the large multicenter approach and the quality in data accrual, our study had relevant limitations. First, StuDoQ|Pancreas captures data from exclusively surgically treated patients, why data composition hampers a more comprehensive picture of the entire PCL spectrum. For example, a number of patients initially referred to participating centers or a number of patients under surveillance remain elusive. Additionally, molecular and radiological data are largely missing despite their eminent role in PCL discrimination. Overall, our data do not provide sufficient information regarding the preoperative diagnosis of (malignant) PCL but may support previous evidence with multicenter data. In particular, the evaluation of postoperative risks as a function of patient-related and intraoperative factors is the strength of the registry and the present work. While there is a risk of bias regarding potential heterogeneity in data entry, the comprehensive approach across multiple centers and surgeons is a key strength of data (-analysis).

## Conclusion

The present study provides in-depth insight into the current surgical treatment of PCL in Germany. Although the treatment of PCL by oncologic resection is consistent with international guidelines, the current evaluation highlights the potential benefit of parenchymal-sparing resections. Also, given the low rate of malignant histology in our cohort, the extent of resection in PCL must always be critically discussed. There appears to be no uniform approach to both the access route and lymphadenectomy, which is reflected by the lack of recommendations in the guidelines. Surgically treated patients with PCL are at high perioperative risk. Especially in patients with identified risk factors (i.e., liver cirrhosis, age, obesity), the guideline recommendation for multidisciplinary evaluation should therefore be applied. The interdisciplinary treatment of PCL is steadily increasing in complexity and requires state-of-the-art resources such as MRI, EUS, and molecular analysis. We demonstrate that large multicenter efforts have the potential to identify clinically relevant preoperative factors that support surgical decision-making in PCL and could serve as a framework for conducting prospective future research (i.e., implementation of radiologic and molecular data). Ultimately, the concise balance of physical capabilities of a patient harboring a PCL against the need for surgery will result in improved personalized medicine.

### Supplementary Information

Below is the link to the electronic supplementary material.

Supplementary file1 (XLSX 27 KB)

## Data Availability

The data that support the findings of this study are available from the corresponding author, HM, upon reasonable request.
